# The antiretroviral efficacy of highly active antiretroviral therapy and plasma nevirapine concentrations in HIV-TB co-infected Indian patients receiving rifampicin based antituberculosis treatment

**DOI:** 10.1186/1742-6405-8-41

**Published:** 2011-11-02

**Authors:** Sanjeev Sinha, Sahajal Dhooria, Sanjiv Kumar, Nipam Shah, T Velpandian, AK Ravi, Narendra Kumar, Hafeez Ahmad, Akshat Bhargwa, Karan Chug, Naresh Bumma, Rahul Chandrashekhar, Meera Ekka, Vishnu Sreenivas, Surendra K Sharma, JC Samantaray, Ronald Mitsuyasu

**Affiliations:** 1 Department of Medicine, All India Institute of Medical Sciences, Ansari Nagar, New Delhi 110029, India; 2 Department of Ocular Pharmacology & Pharmacy, All India Institute of Medical Sciences, Ansari Nagar, New Delhi 110029, India; 3 Department of Microbiology, All India Institute of Medical Sciences, Ansari Nagar, New Delhi 110029, India; 4 Department of Biostatistics, All India Institute of Medical Sciences, Ansari Nagar, New Delhi 110029, India; 5 UCLA Center for Clinical AIDS Research & Education, University of California, 9911 W Pico Blvd Ste 980, Los Angeles, CA 90035, USA

**Keywords:** rifampicin, nevirapine, human immunodeficiency virus (HIV), tuberculosis (TB)

## Abstract

**Background:**

Rifampicin reduces the plasma concentrations of nevirapine in human immunodeficiency virus (HIV) and tuberculosis (TB) co-infected patients, who are administered these drugs concomitantly. We conducted a prospective interventional study to assess the efficacy of nevirapine-containing highly active antiretroviral treatment (HAART) when co-administered with rifampicin-containing antituberculosis treatment (ATT) and also measured plasma nevirapine concentrations in patients receiving such a nevirapine-containing HAART regimen.

**Methods:**

63 cases included antiretroviral treatment naïve HIV-TB co-infected patients with CD4 counts less than 200 cells/mm^3 ^started on rifampicin-containing ATT followed by nevirapine-containing HAART. In control group we included 51 HIV patients without tuberculosis and on nevirapine-containing HAART. They were assessed for clinical and immunological response at the end of 24 and 48 weeks. Plasma nevirapine concentrations were measured at days 14, 28, 42 and 180 of starting HAART.

**Results:**

97 out of 114 (85.1%) patients were alive at the end of 48 weeks. The CD4 cell count showed a mean increase of 108 vs.113 cells/mm3 (p=0.83) at 24 weeks of HAART in cases and controls respectively. Overall, 58.73% patients in cases had viral loads of less than 400 copies/ml at the end of 48 weeks. The mean (± SD) Nevirapine concentrations of cases and control at 14, 28, 42 and 180 days were 2.19 ± 1.49 vs. 3.27 ± 4.95 (p = 0.10), 2.78 ± 1.60 vs. 3.67 ± 3.59 (p = 0.08), 3.06 ± 3.32 vs. 4.04 ± 2.55 (p = 0.10) respectively and 3.04 μg/ml (in cases).

**Conclusions:**

Good immunological and clinical response can be obtained in HIV-TB co-infected patients receiving rifampicin and nevirapine concomitantly despite somewhat lower nevirapine trough concentrations. This suggests that rifampicin-containing ATT may be co administered in resource limited setting with nevirapine-containing HAART regimen without substantial reduction in antiretroviral effectiveness. Larger sample sized studies and longer follow-up are required to identify populations of individuals where the reduction in nevirapine concentration may result in lower ART response or shorter response duration.

## Introduction

There are 33.3 million people living with human immunodeficiency virus/acquired immunodeficiency syndrome (HIV/AIDS) in the world [1]. Out of these, around 40% of patients are co-infected with tuberculosis (henceforth, called HIV-TB co-infected patients), forming a total estimated co-infection prevalence of 13-15 million persons worldwide [2]. As per the latest report by National AIDS Control Organization (NACO), the prevalence of HIV in India is 0.29% with a total burden of 2.27 million HIV-infected patients [3]. NACO has a free antiretroviral therapy (ART) programme in place since April, 2004, which provides antiretroviral drugs in India according to the WHO guidelines [4]. Nevirapine is frequently used in India in HIV/AIDS treatment as a component of first-line regimens, and nevirapine-based fixed-dose combinations (with zidovudine plus lamivudine or stavudine plus lamivudine). These drug combinations are modestly priced, do not require food restrictions, and are given as two tablets twice daily, ensuring good adherence [5-9]. Rifampicin is an important anti- tuberculosis drug and is usually administered for 6 to 8 months along with other anti- tuberculosis medications.

For HIV-TB co-infected patients, the WHO and NACO recommends efavirenz-based ART as rifampicin, which is an essential component of anti-tuberculosis treatment (ATT) reduces the plasma concentration of nevirapine [10]. Also, there is concern about an increased risk of hepatotoxicity as both rifampicin and nevirapine are hepatotoxic. Recent studies have shown that although nevirapine concentrations are lower when it is co-administered with rifampicin, the immunological and virological responses of nevirapine-containing ART have been good [11,12]. There have been, however, notable differences in the effect of rifampicin on nevirapine concentrations in studies reported from different ethnic groups [13-15].

The present study was conducted to explore the efficacy and safety of nevirapine-based ART in HIV-TB co-infected ART-naïve Indian patients who were given rifampicin-based anti-tuberculosis (ATT) concomitantly with HAART. The study also measured their serum nevirapine concentrations and correlated them with the immunological and virological responses to HAART.

## Methods

This was a prospective study conducted at the All India Institute of Medical Sciences (AIIMS), New Delhi between September, 2007 and March, 2011. Patients who tested positive for HIV by ELISA and were ART-naïve and presented with concomitant TB were enrolled as cases. Patients, who tested positive for HIV by ELISA, were ART-naïve and without TB were enrolled as controls. Only patients having CD4 count < 200 cells/mm^3 ^and with normal renal and hepatic function (SGOT and SGPT ≤ 5 × upper normal limit, Serum Bilirubin ≤ 2.5 × upper normal limit and Creatinine ≤ 3 × upper normal limit) were included. The other inclusion criteria were age > 18 years, non-pregnant as confirmed by a negative urine pregnancy test, and absence of concomitant diabetes mellitus. Hepatitis B and C serologies were done and patients testing positive were excluded, as it could have a bearing on hepatotoxicity of study drugs which was one of the outcomes. Also, patients on anti-epileptic drugs, immunosuppressants and other drugs that induce liver microsomal enzyme systems were excluded. HIV infection was documented by licensed ELISA test kit (As per NACO guidelines). CD4/CD8 cell counts were determined by flow- cytometry (BD FACS CALIBUR). Viral load testing was done using AMPLICOR HIV-1 Monitor Test, version 1.5, manufactured by ROCHE Diagnostics. The protocol was approved by the institutional research Ethics Committee of the All India Institute of Medical Sciences, New Delhi. All participants gave signed informed consent to participate in this study.

### Initial evaluation

All patients underwent a detailed physical examination. Their body weight and height were measured and their basal metabolic index (BMI) was calculated. Haemoglobin, complete blood counts, erythrocyte sedimentation rate, fasting blood glucose, renal function tests, liver function tests, serum albumin, serum uric acid and routine urinalysis were done for all patients. In addition, their CD4 counts and plasma HIV viral load were determined at baseline, six months and 12 months.

### Treatment

Cases were started first on anti-tuberculosis treatment (ATT) according to the Revised National Tuberculosis Control Programme (RNTCP) guidelines for directly observed therapy, short-course (DOTS) [16]. After two to eight weeks of ATT, they were started on antiretroviral drug therapy, which consisted of zidovudine, lamivudine and nevirapine (fixed drug combination). The control group was started on ART when CD4 count < 200 cells/mm^3^. Those who had haemoglobin less than 8 g/dl were administered stavudine in place of zidovudine. The doses that were administered were in accordance with the NACO guidelines. Zidovudine was given in a dose of 300 mg twice a day, lamivudine 150 mg twice a day and stavudine 30 mg twice a day. Nevirapine was administered at a dose of 200 mg once a day for the first 14 days (called the lead-in dose) as per NACO guideline, and then the dose was escalated to 200 mg twice a day. The patients were advised to take the drug at 9 am for the first 14 days and at 9 am and 9 pm during the rest of the period of follow up.

### Follow up

Patients were assessed at day 14 after the start of ART, then at day 28, and every 4 weeks thereafter through 48 weeks. A complete haemogram and liver and kidney function tests were obtained at all these visits, and CD4 counts were measured at 8 weeks, 24 weeks and 48 weeks after the start of ART. HIV plasma viral load was measured at baseline, at 24 weeks and at the end of 48 weeks only in the cases. Trough nevirapine concentrations were assessed at day 14, day 28, day 42 and at day 180, 12 hours after the evening dose of nevirapine.

### Outcomes

Vital status, clinical progression, Immunological and virological responses, mortality, and drug toxicity were assessed as outcome measures.

### Definitions

Immunological failure was defined as a fall in CD4 counts to baseline concentrations, a 50% fall from the peak CD4 count during treatment or persistent counts below 100 cells/mm^3 ^at the end of 24 weeks. Disease progression was defined as a new or recurrent WHO stage 4 conditions, after at least 6 months of ART. Virologic response was defined as plasma viral load less than 400 copies/ml after 6 months of ART and drug toxicity was characterized as per the division of AIDS Table for Grading the Severity of Adult and Pediatric Adverse Events, December 2004.

### Measurement of nevirapine concentrations

Blood samples for Nevirapine concentrations measurement were taken 12 hours after drug intake. Each patient was properly counselled about taking the drug in time so that the sample can be drawn exactly at 12 hours. Wherever feasible, the patient was asked to take the drug in front of the research staff and the sample was collected at 12 hours. In others, telephonic conversation was used to ensure that the patient has taken the drug on time. All samples were stored at -80°C until analyzed. At the time of processing of the sample, each plasma sample was allowed to reach room temperature. Nevirapine was procured from Indian Pharmacopoeia Commission IPC, Ghaziabad, India. Tablet Olanzapine (as internal standard) was procured commercially from Sun Pharmaceuticals Ltd., Mumbai, India. Thermo Finnigan High Performance Liquid Chromatographic system (Thermo Electron Corp, USA) with PDA detector controlled by ChromQuest (Ver.4.5) software was used to elude the analyte. Purospher Star RP-C18e, 55 × 4 mm, 3 μ particle (Merck, Germany) was used for analytical separation. Electron spray ionization technique in positive mode was applied using Tubo Ionspray source (ABS Biosystems, USA) in a 4000 Q trap MS/MS (MDS SCIEX, Applied Biosystems). Tandem Mass spectroscopy was controlled using Analyst (Ver.1.4.2) software. The same procedure for nevirapine measurement was used in controls as used in the cases.

Plasma spiking of nevirapine in the concentration of 0.07, 0.14, 0.28, 4.48 and 17.9 ng/ml was prepared by using blank plasma. For this, known amount of the nevirapine and olanzapine was added to blank human plasma (obtained from Blood Bank of AIIMS) and separate calibration curve was made. For the analysis of standard calibration curve, best fit was obtained with the inbuilt algorithm of Analysist. Ver. 1.4.2 was used. Best fit obtained for spiking was subjected to quantify unknown concentration. The best fit plotted the ratio of peak height between analyte and internal standard in "abscissa (Y)" axis and taking the ratio of their concentration in "ordinate (x)" axis.

### Statistical analysis

Data were recorded on a pre-designed data sheet and managed on an 'Excel' spreadsheet. All entries were doubly checked for any possible recording error. Mean, frequency and medians were calculated for all quantitative variables along with the respective standard deviations and Interquartile ranges. Being a pilot study, initially sample size calculation was not planned and data was gathered as per the sample of convenience. On later calculation, sample size required was 127 patients per group for 80% power. All analysis was done by Intention to treat analysis principle. The Mann Whitney U-test was used to compare the mean nevirapine concentrations at day 28 in patients alive at 24 and 48 weeks, and those who died during the study period. The generalised estimation equations were used to find out the predictors of immunological response in terms of the increase in CD4. Statistical analysis was performed using statistical software package STATA version 11.0 [(intercooled version), Stata Corporation, Houston, Texas, USA].

## Results

### Enrolment and disposition of patients

A total of 114 patients (63 in cases and 51 in control group) were eligible and enrolled during the study period. They were followed for a period of 48 weeks after the start of ART. The baseline characteristics of these two groups are summarized in table 1. The median [Interquartile range (IQR)] CD4 count was 127 (16-693) vs. 142 (9 -252); p = 0.36, in cases and controls respectively with no significant difference. The median viral load in cases was 161499 (IQR: 129-6080000) copies/ml. The viral load testing was not done in controls as it is not a routine practice in India under the national programme. The other baseline characteristics, including age and sex distribution, body weight, body mass index, liver function test, were similar between the two groups (p > 0.05 for all). However baseline mean haemoglobin (p = 0.01) and albumin (p = 0.001) were lower in the cases group. A majority of our patients had extra pulmonary TB followed by disseminated TB and pulmonary TB. The dominant mode of TB diagnosis was microbiological and radiological. About 79.3% of the patients (50 out of 63) had their first episode of TB and were put on DOTS category I regimen; the rest received DOTS category II regimen as repeated treatment for TB. The median duration of treatment with rifampicin-based ATT before the start of ART was 35 days (IQR, 26-52).

**Table 1:** Comparison of baseline characteristics in case and control group

Variable	HIV+TB (Cases: N = 63)	HIV+ (Controls: N = 51)	P value
**Age (years) Mean, IOR^a^**	36.5 (24-60)	37.2 (25-57)	0.67
**Gender** **Males** **Females**	49 (77.7%) 14(22.22%)	36(70.6%) 15(29.4%)	0.38
**Weight(Kg)** **(Mean ± S.D.)**	47.58 ± 7.78	51.47 ± 9.58	0.01
**BMI (kg/m^2^)** **(Mean + S.D.)^b^**	17.83 ± 4.44	19.15 ± 4.67	0.20
**CD4 cell count (cells/mm^3^)** **(Median, IQR)**	127 (16-693)	142 (9 -252)	0.36
**Viral Load (copies/ml)** **(Median, IQR)**	161499 (129-6080000)	Not done	-
**Haemoglobin (g/dl)**, **(Mean + S.D.)**	10.2 ± 2.08	11.5 ± 1.53	< 0.01
**Bilirubin (mg/dl)** **(Mean + S.D.)**	0.63 ± 0.17	0.64 ± 0.21	0.93
**AST (I.U)** **(Mean + S.D.)**	47.76 ± 39.37	38.79 ± 21.25	0.22
**ALT (I.U.)** **(Mean + S.D.)**	37.33 ± 32.55	35.26 ± 19.84	0.74
**Albumin (g/dl)** **(Mean + S.D.)**	3.72 ± 0.73	4.26 ± 0.57	< 0.001

^a^Inter Quartile Range

^b^Standard deviation

### Response

**Figure 1:**
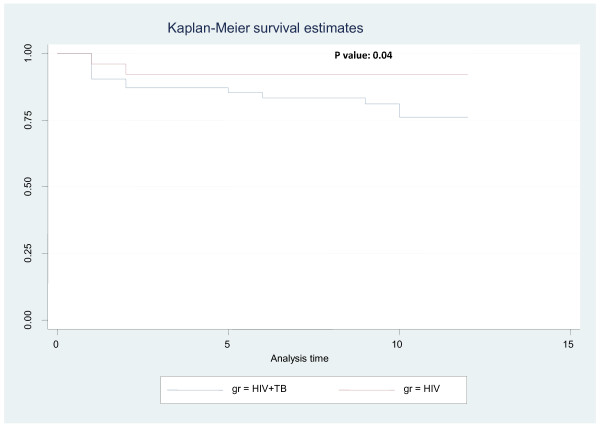
**Kaplan Meier Survival curve in HIV+TB (Cases) and HIV+ (Controls)**.

**Figure 2:**
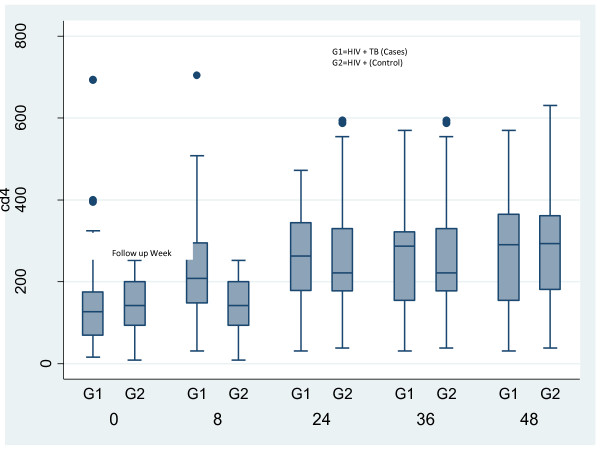
**CD4 cell count at different time points in HIV+TB (Cases) and HIV+ (Controls)**.

The study outcomes are given in detail in table 2. 97 out of 114 (85.1%) patients were alive at the end of 48 weeks. Mortality was higher in cases group (20.63%vs.7.84%), but there was no significant difference stastistically between the groups (p = 0.068). The Kaplan Meier survival curve is depicted in Figure 1. The mean increase in CD4 count was equal in both cases and control at 24 weeks (108.3 vs.112.74 cells/mm3; p = 0.83) and at 48 weeks (128.2 vs.148.4 cells/mm3; p = 0.69) respectively. The CD4 response in cases and controls is shown in Figure 2. The immunological failure was of no significant difference between the two groups (9.5% vs. 3.9%; p = 0.313). The patients also showed a good virological response. Only eight out of the 63 (12.7%) patients, who were alive at the end of the study period, had detectable viral RNA load at the end of 24 weeks. The remaining 87.3% of patients had viral loads of less than 400 copies/ml at the end of 24 weeks. The overall rate of clinical progression was low and it showed no significant difference between the groups (5% vs. 2%, p = 0.30). Regarding general status, the increase in mean weight is equal in both group; it increased to 53.2 vs. 55.7 kg (p = 0.32) at 24 weeks from baseline values of 47.58 vs. 51.47 kg (p = 0.01) in cases and control groups respectively. The mean haemoglobin increased to 12.9 vs.12.5 g/dl (p = 0.33) at 48 weeks from a baseline of 10.2 vs.11.5 g/dl (p = 0.0001) in cases and controls respectively.

**Table 2:** Study outcomes in case and control group

Outcomes	HIV+TB (Cases: n = 63)	HIV+ (Controls: n = 51)	P value
**Vital status at 48 week** **-Weight** **-Hemoglobin**	1. 53.2 ± 8.65 ii. (12.9 ± 1.01	55.7 ± 9.12 12.5 ± 1.52	0.32 0.33
**Mortality**	13 (20.63%)	4 (7.84%)	0.068
**Mean increase in CD4 cell** **count at 24 weeks** **from Baseline**	108.3	112.7	0.83
**Mean increase in CD4 cell** **count at 48 weeks** **from Baseline**	128.2	148.4	0.69
**Viral load suppression at** **24 Weeks** **(≤ 400 copies/ml)**	35 (55.5%)	--	--
**Viral load suppression at 48 Weeks (≤ 400 copies/ml)**	37 (58.73%)	--	--
**Immunological failure**	6 (9.5%)	2 (3.9%)	0.313
**Virological failure**	5 (7.93%)	--	--
**Clinical Progression at** **48 weeks**	6 (9.5%)	2 (3.9%)	0.313

### Nevirapine concentrations

**Figure 3:**
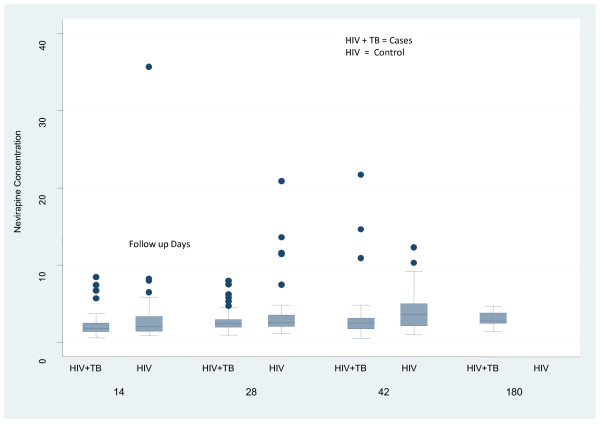
**Plasma Nevirapine concentrations at different time points in HIV+TB (Cases) and HIV+ (Controls)**.

The mean ± SD nevirapine concentrations of cases and control at 14, 28, and 42 days were 2.19 ± 1.49 vs. 3.27 ± 4.95(p = 0.10), 2.78 ± 1.60 vs.3.67 ± 3.59(p = 0.08) and 3.06 ± 3.32 vs. 4.04 ± 2.55 (p = 0.10) respectively and was 3.04 μg/ml at 180 days in cases. The box plot of nevirapine concentrations is shown in Figure 3. The concentration of nevirapine is comparable in both groups with no stastically significant difference. The lowest and the highest nevirapine concentrations at day 28 in our study were 0.92 and 7.95 mcg/ml in cases and 1.1 and 20.9 mcg/ml in controls. The nevirapine trough concentrations at the end of 28 days in the various groups of patients are shown in table 3. The mean nevirapine trough concentration at day 28 was lower in patients who died during the study period than those who were alive at the end of 24 weeks but the difference was not significant (p = 0.85). The nevirapine concentrations at 28 days were also not significantly different between patients who died or had virological failure at the end of 24 weeks and those who were alive with good virological response (p = 0.19). Nevirapine concentration at day 28 was not found to be a significant predictor of the CD4 response at 8 and 24 weeks as estimated by the generalized estimating equations (p = 0.45). There were no sinificant hepatic-toxicities with combined nevirapine/rifampicin.

**Table 3:** Subgroup analysis of HIV+TB (n = 63) against nevirapine trough concentrations at 28 days

**S.N**.	Subgroups	Number of cases	Mean Nevirapine concentrations at day 28 (μg/ml/)	Standard deviation	P value
**1**	HIV+TB (Cases)	63	2.78	1.60	--
**2a**	Cases alive at the end Of 48 weeks	49	2.80	1.53	0.85
**2b**	Cases who died during the study period	13	2.70	1.92	
**3a**	Cases with undetectable viral loads at the end of 24 weeks	28	3.08	1.76	0.19
**3b**	Cases who died or had detectable viral loads at the end of 24 weeks	32	2.52	1.48	
**4a**	Cases with undetectable viral load at the end of 24 weeks	28	3.08	1.76	0.14
**4b**	Cases who were alive but had detectable Viral load at the end of 24 weeks	19	2.40	1.14	

### Adverse events

At the end of 48 weeks, there were a total of 17 deaths in the study population. The exact cause of death was not known for the majority of patients. One of the deaths occurred due to CNS TB, another due to progressive multifocal leucoencephalopathy (PMLE), whereas yet another was due to severe pulmonary TB causing respiratory failure. Two of these patients had shown an increase in CD4 counts at two months. Only two patients had adverse drug reactions. One developed stavudine-induced neuropathy after 80 days of receiving the drug and the other zidovudine-induced anaemia after 62 days. Stavudine was substituted by zidovudine in the first patient and stavudine was substituted to zidovudine in the second. None of the patients in either group had grade 3 or 4 adverse drug reaction.

There were 8 patients in this study who developed immune reconstitution inflammatory syndrome (IRIS). One of the patients had abdominal TB with retroperitoneal lymph nodes. He developed fever and had appearance of new nodes in the cervical area with the start of ART. He was managed with non steroidal anti-inflammatory drugs and improved subsequently. Steroids were not needed for the management of any IRIS case.

## Discussion

This is the first study sponsored by National AIDS Control Organization, Ministry of Health & Family Welfare, Government of India from North India for estimation of plasma nevirapine concentrations in patients receiving nevirapine-containing HAART regimen along with rifampicin-based ATT. Its results indicate that good survival benefit is associated with concomitant administration of ATT and HAART. The mortality was slightly higher in the cases compared to controls. There are confounding factors like absence of tuberculosis in the control group that could be responsible for this difference in mortality. Overall good virological response was obtained in cases. The rate of immunological failure was statistically similar in both the groups. CD4 response (immunological response) is one of the determinants of the effect of HAART [17,18]. Good immunological response was achieved in both cases and control groups in our study. More than half of the patients had CD4 increase greater than 100 at 24 weeks post HAART. Study by Elisa Zaragosa-Macias, et al conducted at Atlanta, Georgia also reported 56% patients had CD4 increase greater than 100 from the baseline at 24 weeks post HAART [18].

Our study shows that good outcomes in terms of vital status and clinical, immunological and virological responses can be obtained in HIV-TB co-infected patients who are administered nevirapine based ART along with rifampicin-based ATT. This is in agreement with earlier studies done by Manosuthi et al [11], Sathia et al [12], and Shipton et al [19]. Manosuthi et al [11] compared patients receiving nevirapine either with or without concomitant administration of rifampicin. They found a non-significant difference between the virological responses in both the groups at 24 weeks. We observed an undetectable viral load in a higher proportion of patients (84% as compared to 72.9% by Manosuthi et al), although the mean nevirapine concentration was lower in our study (2.62 ± 1.61 mcg/ml as compared to 5.40 ± 3.53 mcg/ml) [11]. Sathia et al [12] found that nevirapine concentrations were subtherapeutic in 36% patients receiving rifampicin concomitantly. But they have found that subtherapeutic concentrations were not associated with virological failure. Our findings are not in agreement with those of Boulle et al [20] who have shown that there is a higher risk of virological failure in patients receiving nevirapine with concurrent tuberculosis than those without tuberculosis. The possible explanation for observing a good clinical, immunological and virological response despite lower nevirapine trough concentration is that in our study and the studies by Manosuthi et al [11] and Sathia et al [12] the trough concentrations are much higher than the adjusted median inhibitory concentration (IC_50_) of the drug for the wild type virus. The IC_50 _of free drug nevirapine for the wild type virus is 0.01 mcg/ml. As the drug is 60% protein bound in the plasma and only 40% circulates as free drug, the IC_50 _after adjusting for the degree of protein binding is 0.025 mcg/ml. The mean inhibitory quotient (IQ = Cmin/IC_50_) found in our study was 104 and even for the lowest nevirapine trough concentration observed (0.92 mcg/ml) was 37. Thus the minimum concentration of nevirapine in the plasma of patients in our study was at least 37 times the median inhibitory concentration of nevirapine for the wild type HIV virus. However the minimum IQ for effective therapy has not been defined yet for nevirapine in the international literature and needs further investigation.

The mean Nevirapine trough concentrations were different in the lead in period and during the maintenance dose. The mean nevirapine trough concentrations were sub therapeutic in the lead in period or during the period of dose escalation compared to the trough concentrations at maintenance doses. From pharmacokinetic point of view, Nevirapine administration at dose of 200 mg twice daily would be preferred compared to the Nevirapine 200 mg single dose as supported by Mohammad Lamorde, et al. [21] However, from the clinical view point, our study does not show any advantage of lead in dose compared to the maintenance dose in view of overall good immunological and virological response. The mean nevirapine concentrations achieved in steady state with administration of 200 mg twice a day of nevirapine concomitantly with rifampicin have differed widely in various studies in the current literature. In a Thai study, Autar et al [13] reported a mean nevirapine concentration of 5.47 ± 2.66 μg/ml in the nevirapine-rifampicin group as compared to 8.72 ± 3.98 μg/ml in the nevirapine only group. In a study done in South Africa, Cohen et al [14] reported trough nevirapine concentrations of 3.2 μg/ml (2.8-4.5) and 4.4 μg/ml (3.6-6.9) during and after rifampicin therapy. In a south Indian study published by Ramachandran et al [15], the mean trough concentration of nevirapine when rifampicin was co-administered was 2.59 + 1.36 μg/ml while without rifampicin co-administration, it was 5.48 + 2.35 μg/ml.

We have observed a nevirapine trough concentration which is very similar to that observed in the only published study done on Indian subjects as cited above [15]. It is of note that the studies mentioned above have been done in different ethnic populations. It is known that there are differences in drug disposition and response in different ethnic populations [22,23]. This might be of even greater importance for drugs like nevirapine which are metabolized by liver microsomal enzymes. Nevirapine is principally metabolized by CYP3A4 although other cytochrome systems are also important. Rifampicin is a potent inducer of CYP3A4. It is assumed that it is through the induction of this enzyme system that rifampicin decreases the plasma concentrations of nevirapine. It is widely known that cytochrome P450 enzymes show different distributions of gene polymorphisms in different ethnic populations. This polymorphism may be responsible for the widely different nevirapine concentration in different ethnic populations.

There are several limitations of the present study. Being the sample of convenience, the sample size is small, and the calculated power of the study is 42% for the observed difference of 0.89 μg/dL (p = 0.08). For 80% power the sample size requirement was 127 per group for the observed difference. Also, viral load of controls could not be done and are not available for comparison. However, we came up with some novel findings. To summarise, rifampicin-containing ATT may be co administered with nevirapine-containing HAART regimen, especially in resource limited setting, without substantial reduction in the antiretroviral effectiveness. The nevirapine-containing HAART regimen is much cheaper alternate treatment for HIV and TB coinfection as compare to efavirenz-containing HAART regimen. The studies with larger sample size and longer follow-up will be more helpful to identify the individuals who have reduction in nevirapine concentrations that may result in lower ART response or shorter response duration.

## Competing interests

The authors declare that they have no competing interests.

## Authors' contributions

SS provided inputs to the study design, helped in data analysis and interpretation, wrote the manuscript, and did final editing. SD, NS, AB, KC, NB, RCS, ME and SKS reviewed literature, and helped in interpreting data and writing the manuscript. SK, TV and AKR conducted laboratory tests for nevirapine levels. HA, NK and JCS conducted laboratory tests for CD4 cell count and plasma viral load. VS did data analysis. RLM edited the manuscript. All authors approved and read the final manuscript.
